# Two Cases of Ovarian Dropsy Successfully Treated

**Published:** 1858-05

**Authors:** J. H. Brower

**Affiliations:** Lawrenceburgh, Indiana


ARTICLE V.

### TWO CASES OF OVARIAN DROPSY SUCCESSFULLY TREATED.

BY J. H. BROWER, M.D., LAWRENCEBURGH, INDIANA.

The treatment of ovarian dropsy, by *tapping with pressure*, with a view to radical cure, has been presented to the profession by Mr. J. B. Brown, surgeon-accoucheur of London, in a valuable and lucid summary, of extreme importance to the profession. In the selection of cases, to which Mr. B.'s mode of treatment is specially applicable, a correct diagnosis is of the first importance. He considers unilocular cysts without adhesions, and with clear and not albuminous contents, as presenting the most favorable cases, especially where time and the condition of the patient admit of the persevering application of pressure; but that even cases of multilocular disease and others, where adhesions exist, may be materially benefited or retarded in their growth by pressure.

In tapping the ovary, Mr. B. lays the patient on her side, and prefers a large instrument, and insists that tapping with pressure should always be combined, both as a matter of precaution, when the origin of the cyst is obscure, and as affording increased probability of cure in any case. Compresses of lint or linen should be so arranged as to present a convex surface, adapted as nicely as possible to the concavity of the pelvis; over these, straps of adhesive plaster should be applied so as to embrace the spine, meeting and crossing in front, and be extended from the vertebral articulation of the eighth rib to the sacrum. Over this strapping, a broad flannel roller, or a band with strings and loops which tie or lace in front, may be applied. A strap around each thigh will prevent them from slipping upwards. The bandages and compresses will require occasional adjustment, lest by an unequal pressure, the bowels or bladder may be subjected to inconvenience, and the crest of the ilium should be protected by some soft material.

The effect of pressure *before* tapping is fourfold in its operation. It sometimes retards the filling of the cyst; it may prevent the increase of the tumor; it sometimes brings about absorption of the whole contents; or lastly, it may produce a rupture of the cyst into the vagina, rectum, or peritoneum. *After* tapping, pressure tends to prevent the refilling of the cyst, probably, by compressing mechanically the bloodvessels which supply the fluid. The use of pressure is countenanced by its known good results in dispersing various tumors, or in arresting their growth, as in hydrocele, by strapping the scrotum. When tapping with pressure is resorted to as a means of cure, or even with the view only of retarding the progress of ovarian dropsy, medicines to stimulate the functions of the various abdominal organs, to correct faulty secretions, and generally to improve the health and strength, should also be administered.

The results of this plan of treatment, in a considerable number of cases reported by Mr. Brown, and others, have been extremely favorable, and should induce us to give it a trial. A case of unilocular encysted ovarian dropsy, in the practice of Dr. D. J. Jessup, of Ohio county, with whom I was associated in its treatment, and in which the above process of operation and management was faithfully carried out, ten pints of clear fluid have been drawn off by the trocar under his supervision, has resulted in a radical cure of the ovarian disease and restoration of general health, six months having elapsed without any appearance of a return of the disease. In 1845, a case of a similar character, with the exception, that two distinct cysts occupied the right hypochondriac region, occurred in the practice of Dr. R. H. Torbet, of Dearborn county, upon which I practised the same operation, and after treatment, both cysts having been tapped at one time; after the evacuation of the fluid, amounting in both cysts to nine pints of turbid and albuminous fluid, firm and continuous pressure, by abdominal belts and compresses, was made; in the course of the first forty-eight hours, severe peritonitis set in, which was promptly subdued by venesection and calomel with opium, and with no other untoward event, the case progressed to a final and radical cure, the patient now enjoying perfect health. In both of these cases, iodine frictions over the abdominal surface, in connection with iron and hydriod. potass. internally, seemed materially to aid the cure.

